# Late Complications of COVID-19; a Systematic Review of Current Evidence

**DOI:** 10.22037/aaem.v9i1.1058

**Published:** 2021-01-20

**Authors:** SeyedAhmad SeyedAlinaghi, Amir Masoud Afsahi, Mehrzad MohsseniPour, Farzane Behnezhad, Mohammad Amin Salehi, Alireza Barzegary, Pegah Mirzapour, Esmaeil Mehraeen, Omid Dadras

**Affiliations:** 1 Iranian Research Center for HIV/AIDS, Iranian Institute for Reduction of High Risk Behaviors, Tehran University of Medical Sciences, Tehran, Iran.; 2 Department of Radiology, School of Medicine, University of California, San Diego (UCSD), California, USA.; 3 Department of Virology, School of Public Health, Tehran University of Medical Sciences, Tehran, Iran.; 4 School of medicine, Islamic Azad University, Tehran, Iran.; 5 Department of Health Information Technology, Khalkhal University of Medical Sciences, Khalkhal, Iran.; 6 Department of Global Health and Socioepidemiology, Graduate School of Medicine, Kyoto University, Kyoto. Japan.

**Keywords:** Long Term Adverse Effects, Late Onset Disorders, COVID-19, SARS-CoV-2, post-acute COVID-19 syndrome

## Introduction

### Introduction:

COVID-19 is a new rapidly spreading epidemic. The symptoms of this disease could be diverse as the virus can affect any organ in the body of an infected person. This study aimed to investigate the available evidence for long-term complications of COVID-19.

### Methods:

 This study was a systematic review of current evidence conducted in November 2020 to investigate probable late and long-term complications of COVID-19. We performed a systematic search, using the keywords, in online databases including PubMed, Scopus, Science Direct, Up to Date, and Web of Science, to find papers published from December 2019 to October 2020. Peer-reviewed original papers published in English, which met the eligibility criteria were included in the final report. Addressing non-human studies, unavailability of the full-text document, and duplicated results in databases, were characteristics that led to exclusion of the papers from review. 

### Results:

The full-texts of 65 articles have been reviewed. We identified 10 potential late complications of COVID-19. A review of studies showed that lung injuries (n=31), venous/arterial thrombosis (n=28), heart injuries (n=26), cardiac/brain stroke (n=23), and neurological injuries (n=20) are the most frequent late complications of COVID-19.

### Conclusion:

 Since we are still at the early stages of the COVID-19 epidemic, it is too soon to predict what long-term complications are likely to appear in the survivors of the disease in years after recovery. Furthermore, the complexity of COVID-19 behaviors and targets in the human body creates uncertainty in anticipating long-term complications. 

## Introduction

Coronavirus disease 2019 (COVID-19) is an extremely contagious infectious disease caused by SARS-CoV-2 (1). COVID-19 infection was first reported in Wuhan, China, and spread quickly and turned into an unprecedented global pandemic (2-5). 

The novel coronavirus affects not only the respiratory tract, but also other organs in the human body. COVID-19 could cause injuries in the lungs, liver, kidney, heart, vessels, and other organs (6). Respiratory failure and acute respiratory distress syndrome (ARDS) are the most common complications of severe COVID-19 infection; the majority of hospitalized COVID-19 patients suffer from severe lung injuries and fatal multi-organ failure as well as hemolytic anemia. However; super infection, acute liver, kidney, and cardiac injuries, shock, and hypoxic encephalopathy are less common symptoms (7-9). Some COVID-19 patients may also present signs of tissue damage including rhabdomyolysis or hemoptysis, which lead to cellular injury, release of heme proteins, and collection of heme in body tissues (10).

SARS-CoV-2 usually affects the respiratory system (11), nervous system involvement has also been reported in some recent studies among patients with COVID-19 (12). Coronaviruses can attack the neural tissue including microglia, astrocytes, and macrophages, and cause nerve injury through direct nerve infection (13). The nervous system injuries could manifest as headache, dizziness, seizure, impaired consciousness, acute cerebrovascular disease, and ataxia. The virus could also affect the peripheral nervous system (PNS) and cause olfactory dysfunction, dysgeusia, vision impairment, and neuropathic pain (12, 13). 

COVID‐19 could also cause cardiac injuries such as cardiomyopathy and conduction system malfunction. Studies suggest the direct involvement of cardiac muscles in some patients (4, 14, 15). Generally, infectious myocarditis is the most common cardiac complication of COVID-19 infection. SARS-CoV-2 uses the angiotensin-converting enzyme 2 (ACE2) receptors to infect host cells, through which it can cause pneumonia and myocardial injuries. High expression of ACE2 receptors in the lungs and heart could increase the risk of myocardial injuries in COVID‐19 patients (14). ACE2 is also expressed in the intravascular endothelium, intestinal epithelium, and the kidneys; therefore, these organs could be a target for SARS-CoV-2 infection. Tachyarrhythmia is also a common cardiovascular complication in COVID-19 patients. Electrocardiography and echocardiography could be used in diagnosing and predicting the prognosis in COVID-19 patients (16).

Some COVID-19 patients could suffer from earache that may be a sign of sub-acute thyroiditis. Studies have shown that a few weeks after upper respiratory tract involvement, subacute thyroiditis may occur and it might be a late complication in patients with COVID-19 infection. Therefore, thyroid functions should be checked after discharge in patients with COVID-19 (17, 18). In addition, there is an abnormal rise in various biochemical parameters such as erythrocyte sedimentation rate (ESR), albumin levels, serum ferritin levels, lactate dehydrogenase (LDH) levels, and C-reactive protein (CRP) levels in the infected patients; on the other hand, the hemoglobin levels and lymphocyte count could reduce in these patients. These complications could lead to cytokine storm, causing multiple organ dysfunction (19, 20). 

The coronavirus *pandemic* showed that COVID-19 could affect many organs besides the lungs, like heart and brain, which increases the risk of long-term health problems. There are several ways that the infection can affect someone’s health. Much is still unknown about how COVID-19 will affect people over time. While most patients infected with COVID-19 recover quickly, the potential long-lasting problems caused by COVID-19 make it necessary to look for and study its late complications. This review aims to present a systematic review of late complications of COVID-19 and identify how prevalent these symptoms are and who is most likely to be affected by them. 

## Methods

***Study design and setting***

This study was a systematic review of current evidence conducted in October 2020 and subsequently updated on November 4, 2020. The Preferred Reporting Items for Systematic Reviews and Meta-Analyses (PRISMA) checklist was employed.

***Data sources***

We performed a systematic search using the keywords in the online databases including PubMed, Scopus, Science Direct, Up to Date, and Web of Science. All the related papers and reports published in English from December 2019 through October 2020 were retrieved and then updated in November 2020. Our search strategy in each of the above-mentioned databases included several combinations of keywords in the following orders: 

A. “Coronavirus” OR “COVID-19”OR“SARS-CoV-2” OR “Novel Coronavirus” OR “2019-nCoV" [Title/Abstract]

B. "Clinical characteristics” OR “clinical feature” OR “clinical manifestation" [Title/Abstract]

C. "Consequences” OR “Chronic complications” OR“ Late complications” OR “Long-term effects" [Title/Abstract]

D. [A] AND [B] AND [C]

***Study selection***

The most relevant studies based on titles and abstracts were retrieved by three independent investigators. The full contents of the retrieved papers were reviewed, and the most relevant papers were selected based on the eligibility criteria. The relevant data were extracted and organized in tables. The peer-reviewed original papers published in English that met the eligibility criteria were included in the final report. The exclusion criteria were as follows:

- Papers addressing non-human studies including in vitro investigations or publications concentrating on animal experiments, or discussing COVID-19 in general, without reference to the keywords of this study.
- Unavailability of the full-text document.
- Duplicated results in databases.

***Data extraction***

We used the data extraction sheet (Table 2) to summarize the information of the authors, type of article (e.g., case series), country of origin, study population, and clinical symptoms (late complications in this study). Two independent investigators gathered this information and further organized them in the Tables. All the selected articles were cross-checked by other authors to ensure no duplications or overlap exists in the content.

***Quality assessment***

For bias risk assessment, two independent reviewers rated the quality of included studies by applying the National Institute of Health (NIH) Quality Assessment Tools for Case Series Studies. For this purpose, we have designed a table and evaluated the studies according to NIH questionnaire (Table 1). A third independent investigator was consulted to resolve probable difference of opinions in any case. The full text of select articles was fully read and the key findings were extracted. The final report including the key findings is summarized in Table 2.

## Results

**Figure 1:**
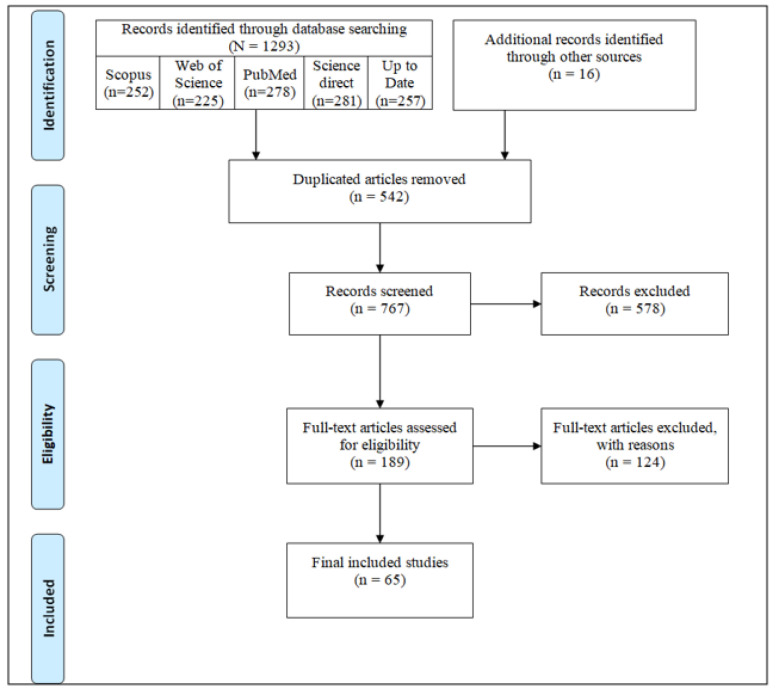
Flow diagram of the selection process of articles identified.

We retrieved 1325 documents using a systematic search strategy. After an initial review of retrieved articles, 542 duplicates were removed, and the titles and abstracts of the remaining 783 articles were reviewed. Applying the selection criteria, 718 articles were excluded, and only 65 articles met the inclusion criteria and were included in the final review (Figure 1). 

We identified 10 potential late complications of COVID-19 including neurological injuries, lung, liver, kidney, and heart injuries, thromboembolism, cardiac/brain stroke, encephalopathy, and psychological distress. Furthermore, some studies have pointed out other complications such as hypoproteinemia, septic shock, and multiple organ dysfunction syndromes (Table 1).

**Figure 2:**
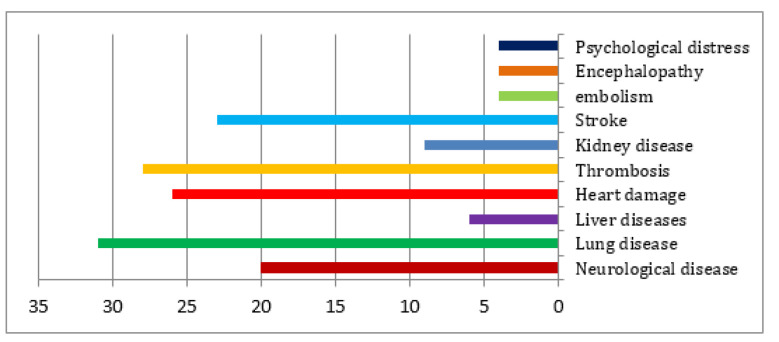
Frequency of identified late complications of COVID-19.

Review of studies showed that lung injuries (n=31), venous/arterial thrombosis (n=28), heart injuries (n=26), cardiac/brain stroke (n=23), and neurological injuries (n=20) were the most frequent late complications of COVID-19. Frequencies of identified late complications of COVID-19 are demonstrated in Figure 2.

## Discussion

One of the most important unknown features of COVID-19 is the duration of symptoms. In the early stages of the disease, the experts believed that the recovery time for mild cases of COVID-19 is 1-2 weeks (21). However, later in many patients, the symptoms lasted for 8 to 10 weeks or even longer, and in some cases, the initial symptoms were replaced by long-term complications such as lung or cardiac injuries (22). Since COVID-19 is a novel virus, there are limited studies about its late complications; it is just a few months since the recovery of the first patients in China. However, the available evidence suggests that the coronavirus can cause long-term complications in an infected person as it may cause major injuries to the heart, kidneys, brain, and even blood vessels (6, 10, 23, 24).

**Table 1:** Quality ratings of included studies based on NIH quality assessment (QA) tool for case series studies

First Author	^*^Question									Rating	
	1	2	3	4	5	6	7	8	9	# 1	# 2
Ali Sepehrinezhad (21)	Yes	Yes	CD	CD	NA	Yes	CD	NA	Yes	Fair	Fair
Filatov A (22)	Yes	Yes	CD	CD	NA	Yes	CD	Yes	Yes	Fair	Fair
Helms J (25)	Yes	Yes	NA	CD	NA	Yes	CD	Yes	Yes	Fair	Fair
Heneka MT (2)	Yes	Yes	CD	CD	NA	Yes	CD	Yes	Yes	Fair	Fair
Kochi AN (26)	Yes	Yes	CD	CD	NA	Yes	CD	NA	Yes	Fair	Fair
Klok FA (27)	Yes	Yes	NA	CD	NA	Yes	CD	NA	Yes	Fair	Fair
Klok FA (30)	Yes	Yes	NR	NA	NA	Yes	CD	Yes	Yes	Fair	Fair
Klok FA(30)	Yes	Yes	CD	CD	NA	Yes	CD	NA	Yes	Fair	Fair
Kunutsor SK(44)	Yes	Yes	CD	NA	NA	Yes	CD	Yes	Yes	Fair	Fair
Landi A (28)	Yes	Yes	CD	NA	NA	Yes	CD	Yes	Yes	Fair	Fair
Lazar HL (45)	Yes	Yes	CD	NA	NA	Yes	CD	Yes	Yes	Fair	Fair
Lee M (46)	Yes	Yes	NA	CD	NA	Yes	CD	Yes	Yes	Fair	Fair
Liabeuf S(47)	Yes	Yes	CD	CD	NA	Yes	CD	Yes	Yes	Fair	Fair
Liu B (48)	Yes	Yes	NA	CD	NA	Yes	CD	Yes	Yes	Fair	Fair
Lorenzo-Villalba N(49)	Yes	Yes	NR	CD	NA	Yes	CD	Yes	Yes	Fair	Fair
Loungani RS(50)	Yes	Yes	NR	CD	NA	Yes	CD	Yes	Yes	Fair	Fair
Lodigiani C(34)	Yes	Yes	CD	CD	NA	Yes	CD	Yes	Yes	Fair	Fair
Long B (51)	Yes	Yes	CD	CD	NA	Yes	CD	Yes	Yes	Fair	Fair
Lopez M (52)	Yes	Yes	CD	CD	NA	Yes	CD	Yes	Yes	Fair	Fair
Ma J (53)	Yes	Yes	CD	NA	NA	Yes	CD	Yes	Yes	Fair	Fair
Ma L (54)	Yes	Yes	CD	NA	NA	Yes	CD	NA	Yes	Fair	Fair
Mao L(43)	Yes	Yes	CD	NA	NA	Yes	CD	NA	Yes	Fair	Fair
Mauro V(55)	Yes	Yes	NA	CD	NA	Yes	CD	Yes	Yes	Fair	Fair
Mendoza-Pinto C(56)	Yes	Yes	NR	CD	NA	Yes	CD	Yes	Yes	Fair	Fair
Nobile B (57)	Yes	Yes	CD	CD	NA	Yes	CD	Yes	Yes	Fair	Fair
Nogueira MS (29)	Yes	Yes	CD	CD	NA	Yes	CD	Yes	Yes	Fair	Fair
Orsi FA(33)	Yes	Yes	NA	CD	NA	Yes	CD	Yes	Yes	Fair	Fair
Oudkerk M(58)	Yes	Yes	NA	NA	NA	Yes	CD	Yes	Yes	Fair	Fair
Palmer K (59)	Yes	Yes	NA	NA	NA	Yes	CD	Yes	Yes	Fair	Fair
Poggiali E (35)	Yes	Yes	NA	CD	NA	Yes	CD	Yes	Yes	Fair	Fair
Parry AH (60)	Yes	Yes	NA	CD	NA	Yes	CD	Yes	Yes	Fair	Fair
Patel VG (61)	Yes	Yes	NA	NA	NA	Yes	CD	NA	Yes	Fair	Fair
Paul P (62)	Yes	Yes	CD	NA	NA	Yes	CD	Yes	Yes	Fair	Fair
Paybast S (42)	Yes	Yes	CD	NA	NA	Yes	CD	Yes	Yes	Fair	Fair
Pryce-Roberts A(38)	Yes	Yes	NA	CD	NA	Yes	CD	Yes	Yes	Fair	Fair
Puntmann VO(37)	Yes	Yes	NA	CD	NA	Yes	CD	Yes	Yes	Fair	Fair
Rey JR (63)	Yes	Yes	NA	CD	NA	Yes	CD	Yes	Yes	Fair	Fair
Roche JA(64)	Yes	Yes	CD	CD	NA	Yes	CD	Yes	Yes	Fair	Fair
Rosen RJ(65)	Yes	Yes	NA	NA	NA	Yes	CD	Yes	Yes	Fair	Fair
Saban-Ruiz J (66)	Yes	Yes	NR	CD	NA	Yes	CD	Yes	Yes	Fair	Fair
Sheraton M (39)	Yes	Yes	CD	CD	NA	Yes	CD	Yes	Yes	Fair	Fair
Siguret V (67)	Yes	Yes	CD	CD	NA	Yes	CD	Yes	Yes	Fair	Fair
Silingardi R (68)	Yes	Yes	CD	CD	NA	Yes	CD	Yes	Yes	Fair	Fair
Silverman – Chen Lin DA (69)	Yes	Yes	CD	NA	NA	Yes	CD	Yes	Yes	Fair	Fair
Singh Y (23)	Yes	Yes	NA	NA	NA	Yes	CD	Yes	Yes	Fair	Fair
Stevens DV (70)	Yes	Yes	NA	NA	NA	Yes	CD	Yes	Yes	Fair	Fair
Strafella C (40)	Yes	Yes	NA	NA	NA	Yes	CD	Yes	Yes	Fair	Fair
Tian D (71)	Yes	Yes	NA	NA	NA	Yes	CD	Yes	Yes	Fair	Fair
Thomas W (72)	Yes	Yes	CD	CD	NA	Yes	CD	Yes	Yes	Fair	Fair
Terpos E (73)	Yes	Yes	NR	CD	NA	Yes	CD	Yes	Yes	Fair	Fair
Varatharaj A (41)	Yes	Yes	NA	CD	NA	Yes	CD	Yes	Yes	Fair	Fair
Varatharajah N (24)	Yes	Yes	NA	CD	NA	Yes	CD	Yes	Yes	Fair	Fair
Wagener F (10)	Yes	Yes	CD	CD	NA	Yes	CD	NA	Yes	Fair	Fair
Wang X (6)	Yes	Yes	NA	NA	NA	Yes	CD	Yes	Yes	Fair	Fair
Zhu H (74)	Yes	Yes	CD	NA	NA	Yes	CD	Yes	Yes	Fair	Fair
Abboud H (75)	Yes	Yes	CD	CD	NA	Yes	CD	Yes	Yes	Fair	Fair
Khan S (76)	Yes	Yes	CD	CD	NA	Yes	CD	Yes	Yes	Fair	Fair
Khandait H (77)	Yes	Yes	CD	CD	NA	Yes	CD	Yes	Yes	Fair	Fair
Msigwa S S(78)	Yes	Yes	NA	CD	NA	Yes	CD	Yes	Yes	Fair	Fair
Sheikh A B (79)	Yes	Yes	NA	CD	NA	Yes	CD	Yes	Yes	Fair	Fair
Siripanthong B (80)	Yes	Yes	NA	NA	NA	Yes	CD	Yes	Yes	Fair	Fair
Vonck K (81)	Yes	Yes	CD	NA	NA	Yes	CD	Yes	Yes	Fair	Fair
Wijeratne T (82)	Yes	Yes	NA	NA	NA	Yes	CD	Yes	Yes	Fair	Fair
Yachou Y (83)	Yes	Yes	CD	NA	NA	Yes	CD	Yes	Yes	Fair	Fair
Zaim S (84)	Yes	Yes	NA	CD	NA	Yes	CD	Yes	Yes	Fair	Fair

NA: not applicable; NIH: National Institutes of Health; NR: not reported; CD: cannot determine

*The NIH Quality Assessment Tool for Case Series Studies contains nine questions: 1 = Was the study question or objective clearly stated?, 2 = Was the study population clearly and fully described, including a case definition?, 3 = Were the cases consecutive?, 4 = Were the subjects comparable?, 5 = Was the intervention clearly described?, 6 = Were the outcome measures clearly defined, valid, reliable, and implemented consistently across all study participants?, 7 = Was the length of follow-up adequate?, 8 = Were the statistical methods well-described?, 9 = Were the results well-described?

**Table 2:** Identified late complications of COVID-19

**ID**	**First author **	**Study type**	**Country**	**Study Population**	**Late complications**							
					**Neurologic**	**Lung disease **	**Liver diseases **	**Heart damage**	**Thrombosis**	**Kidneydisease**	**Stroke**	**Other**
1	Ali Sepehrinezhad (21)	Perspective Review	Iran	Patients with neurological complications	√	×	×	×	×	×	×	--
2	Filatov A (22)	Case Report	USA	74-year-old male	√	×	×	×	×	×	×	Encephalopathy
3	Helms J (25)	Editorial	France	COVID19 patients	√	×	×	×	×	×	√	--
4	Heneka MT (2)	Review	Germany	COVID19 patients	√	×	×	×	×	×	×	--
5	Kochi AN (26)	Review	Italy	COVID19 patients	×	√	×	√	×	×	√	--
6	Klok FA (27)	Case-control	Netherlands	COVID19 patients	×	×	×	×	√	×	×	--
7	Klok FA (30)	Case-Control	Netherlands	COVID19 patients	×	×	×	√	√	×	√	Symptomatic acute pulmonary embolism (PE), myocardial infarction or systemic arterial embolism
8	Klok FA(30)	Case-Control	Netherlands	COVID19 patients admitted to the ICU	×	×	×	√	√	×	√	Pulmonary embolism, DVT, Ischemic, systemic arterial embolism
9	Kunutsor SK(44)	Letter to Editor	UK	COVID 19 patients	×	×	√	×	×	×	×	Hypoproteinemia
10	Landi A (28)	Letter to Editor	Italy	COVID-19 patients admitted to ICU	×	√	×	√	√	×	√	--
11	Lazar HL (45)	Commentary	USA	COVID19 patients admitted to the ICU	×	√	×	×	×	×	×	--
12	Lee M (46)	Letter to Editor	China	COVID 19 patients with a history of cardiovascular diseases	×	×	×	√	×	×	×	--
13	Liabeuf S(47)	Cohort	France	hospitalized patients with laboratory-confirmed COVID-19	×	√	×	×	×	√	×	GI damage, septic shock
14	Liu B (48)	Authors Reply	China	COVID19 Patients	×	√	×	×	√	×	×	--
15	Lorenzo-Villalba N(49)	Case Report	France	a patient hospitalized for COVID-19	×	√	×	×	√	×	×	Parotiditis, cutaneous complications such as hemorrhagic bullae with intra-bullae blood clots and dissecting hematomas, Isolated herpetiform lesions, petechial rash
16	Loungani RS(50)	Review	USA	COVID 19 Patients	×	×	×	√	×	×	√	
17	Lodigiani C(34)	Cohort	Italy	COVID19 patients admitted to hospital	×	×	×	√	√	×	√	Acute coronary syndrome (ACS)/myocardial infarction (MI),overt disseminated intravascular coagulation (DIC)
18	Long B (51)	Cohort	USA	COVID19 patients	×	√	×	√	√	×	×	Systematic inflammation, myocardial injury, acute myocardial infarction, dysrhythmias,
19	Lopez M (52)	Review	USA	COVID19 patients	√	√	√	√	√	×	√	Psychological distress
20	Ma J (53)	Letter to Editor	China	three critically ill patients with coronavirus disease 2019 (COVID-19)	√	×	×	×	√	√	√	Multiple organ dysfunction syndrome, dry gangrene, multiple cerebral infarction, refractory disseminated intravascular coagulation (DIC) and pneumothorax
21	Ma L (54)	Review	China	COVID19 patients	×	√	×	√	×	×	√	Pneumonia, persistent hypotension
22	Mao L(43)	Research article	China	Hospitalized Patients With Coronavirus Disease 2019	√	√	×	×	×	×	×	--
23	Mauro V(55)	Point of view	Italy	COVID19 patients	×	×	√	×	×	×	×	--
24	Mendoza-Pinto C(56)	Letter to Editor	Mexico	COVID19 patients	×	×	×	√	√	√	×	Elevated D-dimer, and coagulation abnormalities, catastrophic antiphospholipid syndrome (CAPS), multiple small vessel occlusions, multiorgan system failure
25	Nobile B (57)	Letter to Editor	France	COVID19 patients using Cloripramine	√	√	×	×	×	×	√	Psychological distress, ischemic attacks, leading to brain inflammation and lesions
26	Nogueira MS (29)	Review	Ireland	COVID19 patients	×	√	×	×	×	×	×	Pneumonia, acute respiratory distress syndrome (ARDS) and lymphadenopathy
27	Orsi FA(33)	Review	Brazil	HospitalizedCOVID-19 patients	×	√	×	×	√	×	×	Septic shock or multiple organ dysfunction, ARDS, Hypercoagulability
28	Oudkerk M(58)	Special Report	Netherlands	COVID-19 patients	×	√	×	√	√	√	×	GI damage, vascular endothelial damage
29	Palmer K (59)	Review	Italy	COVID-19 patients with non-communicable disease (NCD)	×	√	×	√	√	×	√	Psychological distress, exacerbated chronic NCD conditions (e.g., asthma, chronic obstructive, congestive cardiac failure)
30	Poggiali E (35)	Case Reports	Italy	An 82-year-old woman, A 64-year-old man	×	√	×	×	√	×	×	Venous thromboembolism, Deep Pulmonary Embolism
31	Parry AH (60)	Letter to Editor	India	COVID-19 patients with pneumonia	×	√	×	×	√	×	×	Diffuse alveolar damage, acute respiratory distress syndrome, pulmonary vascular damage, PTE
32	Patel VG (61)	Letter to Editor-Cohort	USA	COVID-19 patients with prostate cancer	×	√	×	×	×	×	×	--
33	Paul P (62)	Letter to Editor	India	COVID-19 patients	×	√	×	√	×	×	×	ARDS, pneumonia, multiple organ failure, infective myocarditis
34	Paybast S (42)	Review	Iran	COVID-19 patients	√	√	×	×	×	√	√	GI disease, Intracranial hemorrhage, hyposmia and hypogeusia, disorientation, third nerve palsy
35	Pryce-Roberts A(38)	Review	UK	COVID-19 patients	√	×	×	×	×	×	√	Dysgeusia, hyposmia, disorientation, encephalitis, meningoencephalitis, and encephalopathy
36	Puntmann VO(37)	Clinical trial	Germany	Patients Recently Recovered From COVID-19	×	×	×	√	×	×	×	--
37	Rey JR (63)	Letter to editor	Spain	patients attended due to COVID-19	×	√	×	√	√	×	√	Acute coronary syndrome
38	Roche JA(64)	Hypothesis	USA	COVID 19 patients with deregulated BK signaling	×	√	×	×	×	×	×	--
39	Rosen RJ(65)	Letter to editor	USA		×	×	×	×	√	×	√	--
40	Saban-Ruiz J (66)	Review	Spain	Cardiometabolic health/medicine	×	×	×	√	×	×	√	--
41	Sheraton M (39)	Review	USA	Patients with neurological complications	√	×	×	×	×	×	×	Guillain-Barre syndrome
42	Siguret V (67)	Letter to the editor	France	Thrombotic complications in critically ill COVID-19	×	×	×	×	√	×	√	--
43	Silingardi R (68)	Letter to the editor	Italy	Acute limb ischemia in COVID-19 patients	×	×	×	×	√	×	×	Acute limb ischemia-Pulmonary Embolism-Aortic floating thrombus
44	Silverman – Chen Lin DA (69)	Review	USA	COVID-19 patients	×	√	×	×	×	×	×	--
45	Singh Y (23)	Letter to the editor	India	Cellular metabolism mediated complications in COVID-19infection	√	√	×	√	×	√	√	Cell death triggered by ferroptotic stress
46	Stevens DV (70)	Case-Study	USA	Complications of Orbital Emphysema in a COVID-19 Patient	×	√	×	×	√	×	×	--
47	Strafella C (40)	Analytic	Italy	Analysis of ACE2 Genetic Variability Among Populations	√	√	×	√	×	√	×	Sepsis
48	Tian D (71)	Review	China	COVID-19 patients	×	×	√	×	×	×	×	--
49	Thomas W (72)	Letter to the editor	United Kingdom	Thrombotic complication of a patient with COVID-19	×	×	×	×	√	×	√	--
50	Terpos E (73)	Review	Greece	Hematologic complications in COVID-19 patients	×	×	×	√	√	×	×	--
51	Varatharaj A (41)	Case-control	UK	COVID-19 patients	√	×	×	×	×	×	√	Thrombotic complication of a patient with COVID-19
52	Varatharajah N (24)	Letter to the editor	USA	Microthrombotic complications of patients with COVID-19	×	√	×	×	√	×	×	Hematologic complications in COVID-19 patients
53	Wagener F (10)	Viewpoint	Netherlands	Critically ill COVID-19 patients	×	√	×	×	×	×	×	Coagulation abnormality
54	Wang X (6)	Research article	China	Chronic diseases among patients with COVID-19	×	×	√	√	√	√	×	--
55	Zhu H (74)	Review	USA	patients with COVID-19	×	√	×	√	×	×	×	Coagulopathy-DIC
56	Abboud H (75)	Review	Morocco	patients with COVID-19	√	×	×	×	×	×	×	--
57	Khan S (76)	Review	Malaysia	patients with COVID-19	×	×	×	×	√	×	×	--
58	Khandait H (77)	Research article	India	patients with COVID-19	×	√	×	√	√	×	√	Coagulopathy-DIC-Pulmonary Embolism-Deep vein thrombosis
59	Msigwa S S(78)	Review	China	patients with COVID-19	√	×	×	×	×	×	×	--
60	Sheikh A B (79)	Case-report	USA	56-year-old man with COVID-19	×	×	×	√	√	×	×	--
61	Siripanthong B (80)	Review	UK	patients with COVID-19	×	×	×	√	×	×	×	Myocarditis
62	Vonck K (81)	Review	Belgium	patients with COVID-19	√	×	×	×	×	×	×	central nervous system (CNS) manifestations [dizziness, headache, impaired, consciousness, acute cerebrovascular disease (CVD), ataxia and seizure], cranial and peripheral nervous system manifestations (taste impairment, smell impairment, vision impairment and neuropathy), and skeletal muscular injury manifestations
63	Wijeratne T (82)	Review	Australia	patients with COVID-19	√	×	×	×	×	×	√	Acute ischemic stroke
64	Yachou Y (83)	Review	Russia	patients with COVID-19	√	×	×	×	×	×	×	--
65	Zaim S (84)	Review	UK	patients with COVID-19	√	√	√	√	×	√	×	DIC

DVT: Deep vein thrombosis; GI: gastrointestinal; PTE: pulmonary thromboembolism; DIC: disseminated intravascular coagulation.

The available evidence indicates the recurrence of symptoms in some patients who presented with severe initial symptoms (2, 25, 26). The key question is “what causes the recurrence of symptoms?”. It may be caused by the recurrence or persistence of the primary COVID-19 infection or super infection with another virus or even bacteria due to the compromised immune system (27). In addition, the systemic and multiorgan involvement in advanced phases of COVID-19 pneumonia can cause renal failure, liver dysfunction, thrombocytopenia, and coagulation disorders (28). Therefore, the survivors may present a variety of long-term complications in different organs, including a post-recovery syndrome that doctors call "post-COVID lung disease" (29). By looking at the organs affected during an infection, one can imagine what organs are likely to be affected by long-term complications of COVID-19 infection (30).

The most common long-term complication of COVID-19 is lung disease (8, 29, 31). Most of the COVID-19 patients could be recovered completely except for some minor complications such as cough and shortness of breath. However, a certain proportion of patients have excessive lung damage, and some of them develop pulmonary fibrosis (32). Autopsy studies demonstrated the predominance of microvascular thrombosis in the lungs, coincident with markers of inflammation, which is a hallmark of prolonged infection and sepsis (33). Severe lung involvement in COVID-19 patients could increase the likelihood of progression to chronic lung disease and lead to long-term complications (8, 33).

COVID-19 patients may experience both venous and arterial thrombosis due to severe inflammation and hypoxia, long immobilization, and diffuse intravascular coagulation (27, 28). Klok et al. reported the incidence rate of thrombotic complications to be 31% among ICU patients with severe COVID-19 infection (27). The results of another study reported the high number of arterial and, in particular, venous thrombo-embolic late complications (34). Poggiali et al. described two patients with COVID-19 pneumonia who developed venous thromboembolism and reported hypoxia and sepsis as the potential risk factors for vein thromboembolism (VTE)(35).

Recent studies reported an increased risk of heart failure in COVID-19 patients (26, 28, 30). Moreover, episodes of clinical myocarditis have been observed (15). Heart injuries related to COVID-19 may occur over the course of the disease(36). Late involvement of cardiac muscle has been documented in a study by Puntmannet al. In this study, the researchers investigated the cardiac complications in 100 recovered patients; 78% of patients had cardiac involvement in cardiac magnetic resonance imaging (MRI), 76% had detectable high-sensitivity troponin, and 60% had abnormal native T1 and T2, which indicates the presence of active myocardial (37). Compared to the control group with similar preexisting conditions, left ventricle ejection fraction was lower and the ventricular size was higher in COVID-19 patients. In addition, 32% of patients had late gadolinium enhancement and 22% of them had pericardial involvement (36, 37).

COVID-19 can cause damage to the central nervous system, with potentially long-term consequences (38-41). Late neurological complications of COVID-19, whether caused by the virus or by the triggered inflammation, include decreased awareness and absorption, disturbed memory, and dysfunction of the peripheral nervous system (42). In one study from China, more than a third of hospitalized patients with confirmed COVID-19 had neurological symptoms, including dizziness, headaches, impaired consciousness, vision, taste/smell impairment, and nerve pain. These symptoms were more common in patients with severe disease, where the incidence increased to almost 47 percent (43). Another study in France found neurologic features in 58 of 64 critically ill COVID-19 patients (25).

## Conclusion

Since we are still at the early stages of the COVID-19 epidemic, it is too soon to predict what long-term complications are likely to appear in the survivors of the disease in years after recovery. Furthermore, the complexity of COVID-19 behaviors and variety of its targets in the human body create uncertainty in anticipating long-term complications. However, several ongoing studies are set up to examine the physical, psychological, and socio-economic consequences of the COVID-19.

## Data Availability

The authors stated that all information provided in this article are available.
